# Promoting pharmacovigilance through educational strategies: impact of a national training intervention on the knowledge and practice of healthcare providers in Jordan

**DOI:** 10.1080/20523211.2025.2575828

**Published:** 2025-10-28

**Authors:** Nizar Mahmoud Mhaidat, Sayer Al-Azzam, Jaber Mohammad Jaber, Hayaa Abdallah Banat, Reema Karasneh, Mohammad Araydah, Dana Samih Ahmad, Anwar Al-Sadder, Raneem Saed Nofal, William J. Lattyak, Mamoon A. Aldeyab

**Affiliations:** a Clinical Pharmacy Department, Faculty of Pharmacy, Jordan University of Science and Technology, Irbid, Jordan; b Jordan Food and Drug Administration (JFDA), Amman, Jordan; c Department of Basic Medical Sciences, Faculty of Medicine, Yarmouk University, Irbid, Jordan; d Department of Internal Medicine, Istishari Hospital, Amman, Jordan; e World Health Organization Jordan Country Office, Amman, Jordan; f Department of Pharmacy, School of Applied Sciences, University of Huddersfield, Huddersfield, UK; g Statistical Consulting Department, Scientific Computing Associates Corp, River Forest, IL, USA; h Reading School of Pharmacy, University of Reading, Reading, UK

**Keywords:** Pharmacovigilance, building capacity program, healthcare providers, healthcare system, adverse drug reactions

## Abstract

**Background:**

This study assessed how an educational intervention affected healthcare providers’ knowledge and practice of pharmacovigilance (PV) principles, with an emphasis on enhancing the reporting ADRs.

**Methods:**

In this cross-sectional study, a structured questionnaire was utilised. A pre- and post-educational intervention design was used to assess the influence of a PV workshop on ADRs reporting in Jordan. The PV educational workshop was a one-year interactive session that addressed core PV principles.

**Results:**

A total of 250 healthcare providers participated in the study, including 14 general physicians (5.6%), 15 specialist physicians (6%), 93 pharmacists (37.2%), 51 clinical pharmacists (20.4%), 58 nurses (23.2%), 3 midwives (1.2%), and 16 others (6.4%). A positive trend in participants’ familiarity with the PV term was shown, with 69.6% of respondents expressing improvement post-workshop. After the workshop, more than 70% of participants agreed that the reporting of ADRs increased. The utilisation of electronic forms for reporting ADRs was reported to be increased among 68.4% of participants. Regarding the improvement in the awareness of delayed ADRs, clinical pharmacists had higher knowledge scores (3.04) compared to general physicians (2.50; *p* = 0.041). Clinical pharmacists had a higher practice score (3.92) regarding the frequency of filling up a suspected ADR form compared to nurses (3.53; *p* = 0.042). When comparing the period before to after launching the workshop, the number of reported cases of ADRs increased from 546 to 1060, and the number of reported ADRs increased from 1216 to 1763.

**Conclusion:**

The educational intervention improved healthcare providers’ knowledge and practices related to PV and ADR reporting. These findings highlight the importance of targeted training initiatives in strengthening PV systems and promoting a culture of safety within healthcare settings.

## Background

Pharmacovigilance (PV) is the science and practice of identifying, evaluating, understanding, and preventing side effects or any other medication-related issues in order to reduce risks, maximise benefits, and encourage safe and efficient medication use (ABPI, 2024). In 1968, the World Health Organization (WHO) established the Programme for International Drug Monitoring (PIDM) to systematically detect and collect data on medication-related adverse effects, thereby enabling the timely identification of potential safety concerns associated with medicines (UMC, 2025).

Of note, a robust PV system requires active engagement from all healthcare professionals, including physicians, pharmacists, and nurses (Khan et al., 2022). Previous research has demonstrated promising results regarding the effect of educational interventions on healthcare providers’ knowledge and perception of PV (Abu Farha et al., 2018; El-Dahiyat et al., 2023a; Goel, 2018; Kalikar et al., 2020; Selvan et al., 2016). These intervention have also been shown to effectively enhance ADR reporting among healthcare providers (Lopez-Gonzalez et al., 2015; Selvan et al., 2016). Moreover, a recent systematic review and meta-analysis confirmed that educational programmes significantly improve both PV knowledge and ADR reporting, with workshops demonstrating the highest efficacy in increasing reporting rates (Cervantes-Arellano et al., 2024).

In Jordan, the PV system was introduced in 2002 under the supervision of the Jordan Food and Drug Administration (JFDA), aiming to increase medication safety and advance public health (Banat et al., 2022). Several studies have been conducted in Jordan to evaluate healthcare providers’ understanding, awareness, attitudes, perception, and behaviours regarding PV (Abu Farha et al., 2017; Al Rabayah et al., 2019; Banat et al., 2022; Mukattash et al., 2018; Shroukh et al., 2018). Despite generally positive attitudes toward PV, healthcare providers exhibited limited knowledge, low awareness of PV concepts, and infrequent reporting of adverse drug reactions (ADR) (Abu Farha et al., 2017; Al Rabayah et al., 2019; Alsbou et al., 2017; Banat et al., 2022; Mukattash et al., 2018; Shroukh et al., 2018), highlighting the urgent need for targeted interventions including ongoing awareness sessions and educational workshops to bridge the gap between knowledge, attitude, and practice of ADR reporting (Abu Hammour et al., 2017; El-Dahiyat et al., 2023b).

Research from Jordan showed that more education and training sessions are needed to raise awareness and knowledge of PV, as well as to enhance ADR (Abu Farha et al., 2017; Alsbou et al., 2017; Banat et al., 2022; Mukattash et al., 2018). A national PV programme was launched at the start of 2022 in Jordan, focusing on strengthening the national PV system and building capacity. This initiative involved training individuals across various sectors within the health industry, including governmental bodies, private organisations, the Royal Medical Services, Non-Governmental Organizations (NGOs), and academic institutions. The programme's objectives encompassed enhancing the capacities of healthcare providers, raising awareness about and encouraging the practice of spontaneous ADRs reporting, addressing training gaps brought about during the COVID-19 pandemic, establishing new regional PV centres, revising and updating national legal provisions, regulations, and guidelines, and addressing the issue of under-reporting.

This study aimed to determine the impact of an educational workshop on healthcare providers’ PV knowledge, practice, and ADR reporting rates in Jordan.

## Methods

### Study design

This is a cross-sectional study with a pre- and post-intervention design, in which a structured questionnaire was administered before and after the educational intervention.

### Study population and sampling procedure

The study included healthcare providers (physicians, pharmacists, nurses and midwives) from governmental and private sector hospitals across the country. The sample size was calculated with the Raosoft sample size calculator (Raosoft, 2004) using a 5% margin of error, a confidence level of 95%, a population size of 761 (total number of trained doctors, pharmacists and nurses), and a response distribution of 50%. The calculated sample size was 256. Invitations to participate were sent by the Jordan Food and Drug Administration (JFDA) to participants through WhatsApp, and those who agreed to participate provided informed consent. Of the 761 invited providers, 250 participated, resulting in a response rate of 32.85%.

### Intervention: workshop on PV

A national PV programme was initiated by the JFDA in collaboration with the WHO Country Office (WCO). Between March and December 2022, the programme delivered twenty-two interactive workshops across Jordan, held at regional centers, conference halls, health institutions, and hospitals. The Building Capacity Programme was launched with an inaugural ceremony in Amman under the patronage of the Minister of Health, attended by high-level stakeholders from the Ministry of Health, Royal Medical Services, Jordan University Hospital, Prince Hamzah Hospital, Al Bashir Hospital, King Abdullah University Hospital, Al Karak Government Hospital, and the Patient Coalition Association. Representatives from the University of Jordan, Jordan University of Science and Technology, Mutah University, and the Private Hospital Association were also present. The ceremony featured speeches from the Director General of the JFDA, the Head of the WHO Country Office in Jordan, the Director General of the Royal Medical Services, the presidents of the three universities, the Head of the Private Hospital Association, and the Minister of Health.

Workshops were subsequently conducted in Amman (in a specific Hotel, Jordan University Hospital, Prince Hamzah Hospital, Al Bashir Hospital), Irbid (King Abdullah University Hospital), Al Karak (Al Karak Government Hospital), Aqaba, and at the Al Zaatari and Al Azraq refugee camps. Participants included healthcare professionals from public, private, and Royal Medical Services hospitals. The workshops were funded by the WHO Country Office in Jordan.

The training team comprised experienced pharmacists from the JFDA’s national PV centre, including the Heads of the Rational Drug Use and PV Department and the PV Section. All trainers had over five years of experience in PV, quality and risk management, and PV training.

The standardised agenda included approximately 90 presentations, lectures, profession-specific case studies, and practical demonstrations over 6–7-hour sessions. Content covered the history and principles of PV, ADR mechanisms and classifications, national PV guidelines and regulatory framework, risk management and minimisation, medication errors, signal detection, and use of the web-based ADR reporting system Vigiflow, a web-based system developed by the WHO Collaborating Centre for International Drug Monitoring. Participants practiced ADR reporting using both electronic and hard copy forms, with profession-specific cases integrated – such as medication errors for nurses, causality assessment and seriousness evaluation for physicians, and patient counseling and implementation of risk minimisation measures for pharmacists. Interactive elements, including polls and open discussions, encouraged active engagement and addressed the needs of a diverse audience.

### Questionnaires

The questionnaire was developed via an extensive review of existing literature (Abu Farha et al., 2018; El-Dahiyat et al., 2023a; Ferreira-da-Silva et al., 2023; World Health Organization, 2011; Živanović et al., 2022) and PV expert opinions. Pilot testing was conducted with a small sample to assess clarity, relevance, and comprehensiveness, resulting in further adjustments for optimal alignment with the workshop's objectives. The questionnaire contained four distinct sections. Section A captured demographic details (questions 1–6). Section B focused on trainers’ evaluation (questions 7-9). Section C, questions 10–14 and 56 assessed the overall training programme evaluation, and questions 15–27 and 40–54 explored the impact of the workshop on PV knowledge, questions 28–39 evaluated the changes in PV practices. The questionnaire was administered between 4 September 2023 and 3 October 2023 through online survey administration. All responses were anonymous, and no participant identifiers were collected.

### Reporting of ADR

This study evaluated the impact of the training programme on the reporting of ADRs in Jordan by extracting date from the Jordanian PV database. The web-based PV management system (Vigiflow) was utilised to access all data through JFDA personnel. The extracted data were collected during two periods: pre-programme (March 2021 – February 2022) and post-program (March 2022 – February 2023). Extracted data were: age, gender, completeness score, reporter qualifications, seriousness, seriousness criteria, and the System Organ Classes (SOCs).

### Data analysis

Quantitative data from the survey responses were analysed using descriptive and inferential statistical techniques. Likert scale responses were converted into ordinal rank scores, and mean scores were calculated to summarise participants’ perceptions and levels of agreement. Responses were transformed into quantitative ranks using Likert scales: Section B, Q7 to Q9: (1 = Unacceptable to 5 = Outstanding); Section C, Q15 to Q27: (1 = Stayed the same to 4 = Well improved); Section C, Q28 to Q39: (1 = Greatly decreased to 5 = Greatly increased); Section C, Q40 to Q54: (1 = Strongly disagree to 5 = Strongly agree); and Section C, Q56: (1 = Strongly negative to 5 = Strongly positive). Internal consistency of the survey sections was assessed using Cronbach’s alpha, confirming acceptable reliability of the scales (Supplemental Table S1).

Net Agreement Scores (NAS) were also computed to reflect the balance of positive versus negative responses across items. For sections B and C (Q28 to Q54, and Q56), which have a rank from 1-5, the NAS is computed by subtracting the percentage of question responses that scored 2 or less from the percentage of question responses that scored 4 or more. For section C (Q15 to Q27), which has a rank from 1-4, the NAS is computed by subtracting the percentage of question responses that scored 1 from the percentage of question responses that scored 3 or more. In addition, frequencies and percentages were used to describe the participants’ responses. Changes in knowledge (Q15–27) and practice (Q28–Q39), as well as comparisons across different healthcare professions, were tested using the Wilcoxon Signed-Rank test. Statistical significance was set at *p* ≤ 0.05, and results were presented in matrix form to highlight significant differences between groups. The analysis was done using the SCA Statistical System version 8.2 (Scientific Computing Associates Corp) and R programme version 4.3.2 (R Foundation for Statistical Computing).

## Results

### Socio-demographic characteristics of the study participants

The demographic characteristics and professional profiles of the 250 participating healthcare providers are summarised in Table 1. The majority of the participants were female (71.6%), aged 35–44 years (42%), had 6–10 years of work experience (23.6%), had one Bachelor’s degree (61.6%), and were pharmacists (37.2%).

**Table 1.** Demographic characteristics of healthcare providers.

	Healthcare providers (*n* = 250)
**Gender**	
Male	71 (28.4%)
Female	179 (71.6%)
**Age group**	
18–24	15 (6%)
25–34	74 (29.6%)
35–44	105 (42%)
45–54	52 (20.8%)
55 or over	4 (1.6%)
**Years of work experience**	
None	15 (6%)
Two years or less	15 (6%)
3–5 years	24 (9.6%)
6–10 years	59 (23.6%)
11–15 years	49 (19.6%)
16–20 years	43 (17.2%)
21–25 years	31 (12.4%)
26 or over	14 (5.6%)
**The highest level of education**	
One Bachelor’s Degree	154 (61.6%)
Two Bachelor’s Degrees	25 (10%)
One Master’s Degree	48 (19.2%)
Two Master’s Degrees	15 (6%)
PhD	3 (1.2%)
Professional Certification from an accredited educational institution	5 (2%)
**Current primary position**	
General Physician	14 (5.6%)
Specialist Physician	15 (6%)
Pharmacist	93 (37.2%)
Clinical Pharmacist	51 (20.4%)
Nurse	58 (23.2%)
Midwife	3 (1.2%)
Other	16 (6.4%)

### Overall evaluation of trainers and the training program

Trainers’ technical knowledge received positive evaluation by the participants, with a mean score of 3.18. Around 74% rated it as meeting or exceeding expectations, and 8% rated it as outstanding. Most participants were also satisfied with the trainers’ effective verbal communication (83.2%), with a mean score of 3.22. Furthermore, the assessment of the training programme as a training of trainers achieved a positive evaluation, with a mean score of 3.22 and 83.6% expressing satisfaction (Supplemental Table S2). Overall, 90.8% of participants evaluated the training programme positively, with a mean score of 4.2 (Supplemental Table S3).

### The impact of the workshop on the level of PV knowledge

As presented in Table 2, a positive trend in participants’ familiarity with the term ‘PV' was shown, with 69.6% of respondents expressed improvement or well improvement. Moving to participants’ belief in the essential role of PV in the medication life cycle, 76.8% of participants reported improvement or well improvement.

**Table 2.** The impact of the workshop on the pharmacovigilance knowledge.

Question Number	Questions	Mean	Stayed the same	Slightly Improved	Improved	Well improved	Net Agreement Score
Q15	How would you rate the level of improvement in your familiarity with the term ‘Pharmacovigilance (PV)’?	2.848	21 (8.4%)	55 (22%)	115 (46%)	59 (23.6%)	83.2
Q16	How would you rate the level of improvement in your belief that ‘pharmacovigilance is an essential component of the medication life cycle’?	3.04	13 (5.2%)	45 (18%)	111 (44.4%)	81 (32.4%)	89.6
Q17	How would you rate the level of improvement in your familiarity with the WHO’s Program for International Drug Monitoring (PIDM)?	2.832	19 (7.6%)	62 (24.8%)	111 (44.4%)	58 (23.2%)	84.8
Q18	How would you rate the level of improvement in your familiarity with the Uppsala monitoring center?	2.608	38 (15.2%)	58 (23.2%)	118 (47.2%)	36 (14.4%)	69.6
Q19	How would you rate the level of improvement in your familiarity with the established national PV centre in Jordan?	2.82	17 (6.8%)	58 (23.2%)	128 (51.2%)	47 (18.8%)	86.4
Q20	How would you rate the level of improvement in your awareness that some ADRs can occur even after years of stopping the product and in next generations (Delayed ADRs)?	2.876	18 (7.2%)	55 (22%)	117 (46.8%)	60 (24%)	85.6
Q21	How would you rate the level of improvement in your awareness of the available channels of submitting suspected ADRs reports to the national PV centre (e.g. yellow card (paper form), email, FAX, Telephone, Website, QR Code)?	2.82	22 (8.8%)	57 (22.8%)	115 (46%)	56 (22.4%)	82.4
Q22	How would you rate the level of improvement in your awareness that you can report ADRs in both Arabic and English Languages, as reporting forms support these languages?	2.88	17 (6.8%)	58 (23.2%)	113 (45.2%)	62 (24.8%)	86.4
Q23	How would you rate the level of improvement in your familiarity with the use of ADRs reports and the actions can be mADR based on the analysis of these collected and validated ones?	2.82	21 (8.4%)	56 (22.4%)	120 (48%)	53 (21.2%)	83.2
Q24	How would you rate the level of improvement in your awareness that by regulation, as a healthcare provider, you are responsible of reporting ADRs?	2.832	21 (8.4%)	55 (22%)	119 (47.6%)	55 (22%)	83.2
Q25	How would you rate the level of improvement in your awareness that anyone can directly report ADRs including patients, parents and carers, even you as a health care practitioner, without seeking any higher approvals?	2.844	16 (6.4%)	60 (24%)	121 (48.4%)	53 (21.2%)	87.2
Q26	How would you rate the level of improvement in your belief that reporting suspected ADR to the national PV system may have an influence on the patient's personal, social, and/or economic quality of life?	2.872	13 (5.2%)	61 (24.4%)	121 (48.4%)	55 (22%)	89.6
Q27	How would you rate the level of improvement in your belief that reporting ADRs will help with generating more evidence to ensure better understanding and improvement of the safety profile of a product?	2.964	17 (6.8%)	50 (20%)	108 (43.2%)	75 (30%)	86.4

### The impact of the workshop on PV practice

Participants’ responses concerning PV reporting practices are presented in Table 3. After the workshop, over 70% of participants reported improved practices in several aspects of ADR reporting, including reporting suspected ADRs even in cases of uncertainty regarding the medicine's responsibility (70%), reporting both serious and non-serious ADRs (73.6%), reporting expected ADRs that are known and listed in the product’s leaflet (70.4%), reporting unexpected ADRs that are unknown and not listed in the product’s leaflet (70.8%), application of their knowledge to recognise suspected ADR during practice and regular interactions with patients (74.4%), and reading ADR-related articles (70%).

**Table 3.** The impact of the workshop on the pharmacovigilance practice.

Question Number	Questions	Mean	Decreased	Stayed the same	Increased	Net Agreement Score
Q28	Reporting of suspected ADRs even if you are not sure if the medicine is responsible for the reaction	3.752	13 (5.2%)	62 (24.8%)	175 (70%)	64.8
Q29	Reporting of serious and non-serious ADRs	3.816	11 (4.4%)	55 (22%)	184 (73.6%)	69.2
Q30	Reporting expected ADRs (known and listed in the product’s leaflet)	3.768	13 (5.2%)	61 (24.4%)	176 (70.4%)	65.2
Q31	Reporting unexpected ADRs (unknown ones, not listed in the product’s leaflet)	3.816	10 (4%)	63 (25.2%)	177 (70.8%)	66.8
Q32	Using electronic forms to report ADRs	3.768	15 (6%)	64 (25.6%)	171 (68.4%)	62.4
Q33	Using different available channels (eg, yellow card (paper form), email, FAX, Telephone, Website, QR Code) to report ADRs to the national PV center	3.76	19 (7.6%)	62 (24.8%)	169 (67.6%)	60
Q34	Frequency of filing up a suspected ADR form	3.708	14 (5.6%)	74 (29.6%)	162 (64.8%)	59.2
Q35	Easiness of filling up ADR form	3.756	17 (6.8%)	62 (24.8%)	171 (68.4%)	61.6
Q36	Application of your knowledge to recognise suspected ADR during practice and regular interactions with patients	3.868	12 (4.8%)	52 (20.8%)	186 (74.4%)	69.6
Q37	Reading ADR-related articles	3.788	17 (6.8%)	58 (23.2%)	175 (70%)	63.2
Q38	Attending training programme on ADR reporting	3.764	13 (5.2%)	73 (29.2%)	164 (65.6%)	60.4
Q39	Carrying out research activities related to pharmacovigilance	3.74	15 (6%)	72 (28.8%)	163 (65.2%)	59.2

### Comparison of PV knowledge and practice across healthcare professions

Knowledge mean scores for each knowledge-related question (Q15-Q27; Supplemental Material – Appendix 1) were compared across job functions (Supplemental Tables S4 and S5). Clinical pharmacists (Mean = 3.12 (95% CI: 2.97–3.25)) and pharmacists (Mean = 2.88 (95% CI: 2.72–3.03)) demonstrated higher familiarity with the term ‘PV' (Q15) compared to nurses (Mean = 2.64 (95% CI: 2.48–2.78)), with *p*-values of 0.002 and 0.047, respectively. Pharmacists (Mean = 3.19 (95% CI: 3.05-3.35)) had higher knowledge scores regarding the importance of PV in the medication life cycle (Q16) compared to nurses (Mean = 2.84 (95% CI: 2.69–3.00)) (*p* = 0.006). Regarding the improvement in the awareness of delayed ADRs (Q20), clinical pharmacists (Mean = 3.04 (95% CI: 2.88-3.18)) had higher knowledge scores compared to general physician (Mean = 2.50 (95% CI: 2.30–2.68)) and nurses (Mean = 2.71 (95% CI: 2.55–2.87)) (*p* = 0.041 and 0.025 respectively).

Practice scores for each practice-related question (Q28–Q39; Supplemental Material – Appendix 1) were compared across job functions (Supplemental Tablses S6 and S7). Clinical pharmacists (Mean = 3.92 (95% CI: 3.79–4.06)) reported a higher frequency of filling up a suspected ADR form (Q34) compared to nurses (Mean = 3.53 (95% CI: 3.33–3.74)) (*p* = 0.042). Regarding reading ADR-related articles (Q37), clinical pharmacists (Mean = 4.02 (95% CI: 3.89–4.15)) had higher practice scores than both nurses (Mean = 3.53 (95% CI: 3.32–3.72)) and pharmacists (Mean = 3.74 (95% CI: 3.58–3.88)) (*p* = 0.014 and *p* = 0.044, respectively). Clinical pharmacists (Mean = 3.92 (95% CI: 3.76–4.06)) also attended ADR reporting training programs (Q38) more frequently than nurses (Mean = 3.50 (95% CI: 3.31–3.70)) (*p* = 0.02). Furthermore, clinical pharmacists (Mean = 4.00 (95% CI: 3.85–4.14)) had higher practice scores compared to pharmacists (Mean = 3.63 (95% CI: 3.47–3.79)) regarding carrying out research activities related to PV (Q39) (*p* = 0.015).

### Participants’ evaluation of the training workshop

As demonstrated in Table 4, over 85% of the participants agreed that the knowledge and skills they learned will be helpful to them in their work (89.6%), the workshop enhanced their willingness to provide training and mentor others (85.6%), the training/workshop learning objectives were stated clearly and successfully met (85.6%), the training/workshop was well facilitated (86%), the facilitators made the best use of the time allotted to each session (85.6%), they had ample opportunity to ask questions and receive answers to their questions during the training/workshop (86%), they found the venue and set-up to be comfortable, free of distractions, and conducive to learning (86%), and they would recommend this training/workshop to others (85.2%).

**Table 4.** Participants’ evaluation of the training workshop.

Question Number	Questions	Mean	Disagree	Neutral	Agree	Net Agreement Score
Q40	The knowledge and skills I learned will be helpful to me in my work	4.128	7 (2.8%)	19 (7.6%)	224 (89.6%)	86.8
Q41	The workshop enhanced my willingness to provide training and mentor others	4.024	9 (3.6%)	27 (10.8%)	214 (85.6%)	82
Q42	The workshop improved my capacity to provide training and mentor others	3.98	12 (4.8%)	32 (12.8%)	206 (82.4%)	77.6
Q43	The training/workshop learning objectives were stated clearly and successfully met	4.004	8 (3.2%)	28 (11.2%)	214 (85.6%)	82.4
Q44	The training/workshop was well facilitated	4.04	7 (2.8%)	28 (11.2%)	215 (86%)	83.2
Q45	The answers the facilitator gave to participants’ questions were clear and satisfactory	4.032	9 (3.6%)	30 (12%)	211 (84.4%)	80.8
Q46	The facilitator provided illustrative examples	4.024	9 (3.6%)	31 (12.4%)	210 (84%)	80.4
Q47	The facilitator’s made the best use of the time allotted to each session.	4	9 (3.6%)	27 (10.8%)	214 (85.6%)	82
Q48	I had ample opportunity to ask questions and receive answers to my questions during the training/workshop.	4.044	7 (2.8%)	28 (11.2%)	215 (86%)	83.2
Q49	The training/workshop allowed participants to practice practical skills related to essential concepts.	3.96	11 (4.4%)	38 (15.2%)	201 (80.4%)	76
Q50	The training/workshop was interactive and allowed me to be actively engaged.	3.996	11 (4.4%)	32 (12.8%)	207 (82.8%)	78.4
Q51	The workshop was well organised.	4.028	12 (4.8%)	28 (11.2%)	210 (84%)	79.2
Q52	I found the venue and set-up to be comfortable, free of distractions, and conducive to learning.	4.024	12 (4.8%)	23 (9.2%)	215 (86%)	81.2
Q53	I would recommend this training/workshop to other	4.088	10 (4%)	27 (10.8%)	213 (85.2%)	81.2
Q54	I enjoyed the workshop.	4.08	13 (5.2%)	25 (10%)	212 (84.8%)	79.6

### The impact of the workshop on ADRs reporting rates

This study evaluated the cases in which ADRs were reported between March 2021 and February 2023. A total of 1606 cases were reported to the Jordanian PV database (Table 5). The number of cases reported after launching the workshop was higher than that reported before the workshop (1060 vs 546 cases). The mean age of patients with reported ADR was 41.99 years old, with higher percentage of females compared to males (48.75% vs 35.68%). Approximately one-third of the cases were labelled as serious (35.43%), with similar proportions before and after the workshop (34.62% vs. 35.85%).

**Table 5.** Characteristics of reported ADRs cases.

	Total (*n* = 1606)	Before the workshop (*n* = 546)	During the workshop (*n* = 1060)
**Age**	41.99	41.83	42.07
**Gender**			
Male	557 (34.68%)	176 (32.23%)	381 (35.94%)
Female	783 (48.75%)	262 (47.99%)	521 (49.15%)
**Completeness score**	0.47	0.41	0.44
**Reporter qualification**			
Physician	912 (56.79%)	350 (64.1%)	562 (53.02%)
Pharmacist	412 (25.65%)	92 (16.85%)	320 (30.19%)
Other Health Professionals	304 (18.93%)	114 (20.88%)	190 (17.92%)
Consumer/Non-Health Professional	66 (4.11%)	24 (4.4%)	42 (3.96%)
**Seriousness**	569 (35.43%)	189 (34.62%)	380 (35.85%)
**Seriousness Criteria**			
Caused/prolonged hospitalisation	199 (12.39%)	67 (12.27%)	132 (12.45%)
Death	57 (3.55%)	22 (4.03%)	35 (3.3%)
Life-threatening	53 (3.3%)	17 (3.11%)	36 (3.4%)
Disabling/incapacitating	14 (0.87%)	1 (0.18%)	13 (1.23%)
Congenital anomaly/birth defect	1 (0.06%)		1 (0.09%)
Other medically important conditions	364 (22.67%)	126 (23.08%)	238 (22.45%)
Unknown	1037 (64.57%)	357 (65.38%)	680 (64.15%)

Overall, physicians reported the majority of ADR cases (56.79%), followed by pharmacists (25.65%). Prior to the workshop, physicians reported 64.1% of cases compared to 16.85% reported by pharmacists. Nevertheless, following the workshop, the percentages of cases reported by pharmacists rose to 30.19%, while the percentage reported by physicians decreased to 53.02%.

In total, 2797 ADRs were reported to the Jordanian PV database, with a higher number reported after the workshop compared to before (1,763 vs. 1,216) (Table 6).

**Table 6.** The MedDRA system organ classification of reported ADRs.

	Total (*n* = 2797)	Before the workshop (*n* = 1216)	During the workshop (*n* = 1763)
Product quality and use issues	791 (28.28%)	399 (32.81%)	392 (22.23%)
General disorders and administration condition	332 (11.87%)	117 (9.62%)	215 (12.2%)
Skin and subcutaneous tissue disorders	269 (9.62%)	107 (8.8%)	162 (9.19%)
Gastrointestinal disorders	239 (8.54%)	82 (6.74%)	157 (8.91%)
Nervous system disorders	234 (8.37%)	57 (4.69%)	177 (10.04%)
Respiratory, thoracic, and mediastinal disorders	191 (6.83%)	84 (6.91%)	107 (6.07%)
Blood and lymphatic system disorders	132 (4.72%)	62 (5.1%)	70 (3.97%)
Cardiac disorders	101 (3.61%)	34 (2.8%)	67 (3.8%)
Musculoskeletal and connective tissue disorders	84 (3%)	31 (2.55%)	53 (3.01%)
Infections and infestations	80 (2.86%)	48 (3.95%)	32 (1.82%)
Immune system disorders	78 (2.79%)	28 (2.3%)	50 (2.84%)
Metabolism and nutrition disorders	77 (2.75%)	24 (1.97%)	53 (3.01%)
Neoplasms benign, malignant, and unspecified	59 (2.11%)	28 (2.3%)	31 (1.76%)
Renal and urinary disorders	59 (2.11%)	27 (2.22%)	32 (1.82%)
Eye disorders	47 (1.68%)	17 (1.4%)	30 (1.7%)
Pregnancy, puerperium, and perinatal conditions	46 (1.64%)	11 (0.9%)	35 (1.99%)
Hepatobiliary disorders	36 (1.29%)	6 (0.49%)	30 (1.7%)
Psychiatric disorders	35 (1.25%)	21 (1.73%)	14 (0.79%)
Reproductive system and breast disorders	26 (0.93%)	1 (0.08%)	25 (1.42%)
Death	19 (0.68%)	5 (0.41%)	14 (0.79%)
Injury, poisoning, and procedure complications	17 (0.61%)	1 (0.08%)	16 (0.91%)
Poisonings, toxicities, and procedural complications	11 (0.39%)	11 (0.9%)	0 (0%)
Vascular disorders	8 (0.29%)	8 (0.66%)	0 (0%)
Ear and labyrinth disorders	5 (0.18%)	4 (0.33%)	1 (0.06%)
Coagulation and bleeding disorders	2 (0.07%)	2 (0.16%)	0 (0%)
Oral cavity and digestive system disorders	1 (0.04%)	1 (0.08%)	0 (0%)

## Discussion

This study aimed to assess changes in PV knowledge and practice among healthcare providers after following a targeted PV educational capacity-building programme. The results showed positive evaluation of trainers in relation to their technical expertise and communication skills, improvement in familiarity with national and global PV initiatives, significant increase in knowledge and practice scores, and improvement in ADR reporting rate.

Consistent with the results reported in this study, earlier studies in Nepal and India reported significant improvements in healthcare providers’ knowledge of PV following educational interventions (Panneerselvam et al., 2022; Shenoy et al., 2023; Shrestha et al., 2020). In Brazil, a study found that using case-based discussions and involving multidisciplinary teams resulted in better healthcare professionals’ form-completion skills and knowledge of PV (Varallo et al., 2017). Moreover, the positive impact of the educational intervention on healthcare providers’ familiarity with PV concept and awareness of national and global PV initiatives found in our study align with the findings of several other studies (Abu Farha et al., 2018; Arici et al., 2015; Jha et al., 2017; Opadeyi et al., 2019; Panneerselvam et al., 2022; Shenoy et al., 2023; Shrestha et al., 2020; Varallo et al., 2017).

In the current study, over 70% of participants reported improved practices in several aspects of ADR reporting following the educational intervention. Similar findings were observed in a randomised controlled trial that evaluated the impact of a combined educational seminar and monthly SMS reinforcements on healthcare providers’ PV practice over 12 months. Following the intervention, the study found that 82% of the intervention group had observed an ADR, compared to 73.4% in the control group (Opadeyi et al., 2019). Changes in ADR reporting after training were also observed in Vo et al. (2020) study in which an increase of 12.25 times in ADR reports was observed following targeted training workshops in Vietnam (Vo et al., 2020). Similarly, Al Enazi et al. (2024) reported a statistically significant difference in ADR reporting rate before and after an educational intervention among nurses and pharmacists in Saudi Arabia (30.4% versus 59.6%, *P* < 0.0001) (Al Enazi et al., 2024). These findings highlight the practical impact of education on reporting behaviour.

In the present study, scores of knowledges and practices were compared between different healthcare professional categories. PV knowledge and practice scores were found to be higher among clinical pharmacists and pharmacists than other health professionals. Similar findings were observed in a study that was conducted in Albania, in which pharmacists had better knowledge regarding how to report ADRs (51.43%) compared to physicians (46.88%) and nurses (32.69%) (*P* = 0.018) (Shkreli et al., 2023). The higher performance of pharmacists observed in our study is also consistent with the observations of Mustafa et al. (2021) study, in which pharmacists were found to have significantly better knowledge and practice related to PV than physicians and nurses (*P* < 0.001) (Mustafa et al., 2021). Furthermore, in Al Enazi et al. (2024) study, most reported ADRs were by pharmacists (77.0%), followed by technicians (21.9%) and nurses (1.1%) (Al Enazi et al., 2024). The reason behind this result could be related to pharmacists’ specialised education and consistent exposure to medication-related safety issues (Shkreli et al., 2023).

The results of the current study revealed that the majority of ADR cases were reported by physicians (56.79%) and pharmacists (25.65%). Following the workshop, the percentages of cases reported by pharmacists rose from 16.85% to 30.19%, while that reported by physicians decreased from 64.1% to 53.02%. However, the number of cases reported after the workshop increased among all the participating healthcare providers. This finding suggests that the educational intervention was effective in enhancing ADR reporting, regardless of the percentage of ADR reports submitted by each category of healthcare providers. A cluster-randomised controlled trial reported that ADR reporting has increased by 10-fold after one year of conducting a targeted educational intervention among physicians in Portugal (Figueiras et al., 2006). The intervention also increased the reporting rate for unexpected, serious, high-causality, and new drug-related ADRs, which improved the quality of reports (Figueiras et al., 2006).

One limitation of this study is the potential for bias resulting from the inclusion of healthcare providers from different professional backgrounds, such as physicians, pharmacists, and nurses. While all participants were recruited from similar institutional settings within the same healthcare system and were exposed to a uniform educational intervention, their varying levels of baseline knowledge, clinical responsibilities, and experiences with PV could have influenced the way they engaged with the content and responded to the assessments. To address this, the intervention was designed to focus on PV principles that are broadly applicable across healthcare professions, and all workshops were delivered using a standardised curriculum by the same facilitators. Furthermore, the use of pre – and post-intervention analysis allowed us to compare individual-level changes in knowledge and practice, thereby helping to control for variability across professional groups. Analyses also demonstrated improvements across several domains, suggesting the intervention had a positive impact, considering different professional backgrounds. Future studies may benefit from tailoring educational interventions to the unique needs of different professional groups and evaluating their effectiveness separately to provide more targeted insights.

## Conclusion

This study evaluated a year-long multidisciplinary educational intervention and provided invaluable insights into its influence on healthcare providers’ PV knowledge and practices. The intervention significantly improved participants’ understanding and application of PV principles, while also improving practical performance and ADR reporting rates. Clinical pharmacists and pharmacists were more knowledgeable about PV compared to other job functions. These findings emphasise the value of targeted training initiatives in strengthening PV systems and promoting a culture of safety within healthcare settings, while taking into consideration the necessity of customising these programmes to suit various job functions. The long-term effectiveness of such initiatives and their incorporation into regular professional training should be investigated in future research. Continued investment in such programmes is essential to sustain and further enhance ADR reporting practices among healthcare professionals.

## Supplementary Material

Supplemental Material
