# Supplementary material for: Promoting pharmacovigilance through educational strategies: impact of a national training intervention on the knowledge and practice of healthcare providers in Jordan
Source: J Pharm Policy Pract. 2025 Oct 28;18(1):2575828. doi: 10.1080/20523211.2025.2575828 (PMC12570230; doi:10.1080/20523211.2025.2575828)
Supplement: Supplemental Material [file JPPP_A_2575828_SM7519.docx]

**Supplementary Material- Online Material**

| **Table S1.** Estimated Cronbach’s alpha tests for the administered questionnaire | | |
| --- | --- | --- |
| **Focus Sections** | **Items** | **Cronbach’s Alpha** |
| Section B- Q7 to Q9 | 3 | 0.928 |
| Section C - Q15 to Q27 | 13 | 0.963 |
| Section C - Q28 to Q39 | 12 | 0.963 |
| Section C - Q40 to Q54 | 15 | 0.978 |

| **Table S2.** Evaluation of the trainers and training program | | | | | | | | |
| --- | --- | --- | --- | --- | --- | --- | --- | --- |
| **Question Number** | **Questions** | **Mean** | **Unacceptable** | **Needs Improvement** | **Meets Expectations** | **Exceeds Expectations** | **Outstanding** | **Net Agreement Score** |
| Q7 | How do evaluate trainers in relation to their technical knowledge | 3.18 | 10 (4%) | 35 (14%) | 125 (50%) | 60 (24%) | 20 (8%) | 14 |
| Q8 | How do you evaluate trainers in relation to effective verbal communication? | 3.22 | 8 (3.2%) | 34 (13.6%) | 126 (50.4%) | 59 (23.6%) | 23 (9.2%) | 16 |
| Q9 | After enrollment in the national pharmacovigilance program training session, how do you evaluate this program as training of trainer? | 3.22 | 8 (3.2%) | 33 (13.2%) | 125 (50%) | 59 (23.6%) | 25 (10%) | 17.2 |

| **Table S3.** Overall evaluation of the training program | | | | | | |
| --- | --- | --- | --- | --- | --- | --- |
| **Question Number** | **Questions** | **Mean** | **Negative** | **Neutral** | **Positive** | **Net Agreement Score** |
| Q56 | What is your overall evaluation of the training program? | 4.208 | 0 (0%) | 23 (9.2%) | 227 (90.8%) | 90.8 |

| **Table S4.** Mean knowledge scores across job functions | | | | | |
| --- | --- | --- | --- | --- | --- |
| **JOB** | **Job Function** | **Q** | **mean** | **LCL** | **UCL** |
| 0 | General Physician | Q15 | 2.64 | 2.46 | 2.82 |
| 1 | Specialist Physican | Q15 | 2.87 | 2.69 | 3.03 |
| 2 | Pharmacist | Q15 | 2.88 | 2.72 | 3.03 |
| 3 | Clinical Pharmacist | Q15 | 3.12 | 2.97 | 3.25 |
| 4 | Nurse | Q15 | 2.64 | 2.48 | 2.78 |
| 5 | Midwife | Q15 | 2.33 | 2.09 | 2.57 |
| 6 | Other | Q15 | 2.93 | 2.72 | 3.16 |
| 0 | General Physician | Q16 | 3.00 | 2.85 | 3.14 |
| 1 | Specialist Physican | Q16 | 2.93 | 2.75 | 3.08 |
| 2 | Pharmacist | Q16 | 3.19 | 3.05 | 3.35 |
| 3 | Clinical Pharmacist | Q16 | 3.10 | 2.94 | 3.25 |
| 4 | Nurse | Q16 | 2.84 | 2.69 | 3.00 |
| 5 | Midwife | Q16 | 2.33 | 2.25 | 2.43 |
| 6 | Other | Q16 | 3.07 | 2.84 | 3.28 |
| 0 | General Physician | Q17 | 2.64 | 2.41 | 2.86 |
| 1 | Specialist Physican | Q17 | 2.87 | 2.72 | 3.02 |
| 2 | Pharmacist | Q17 | 2.85 | 2.70 | 3.00 |
| 3 | Clinical Pharmacist | Q17 | 3.00 | 2.85 | 3.15 |
| 4 | Nurse | Q17 | 2.74 | 2.57 | 2.90 |
| 5 | Midwife | Q17 | 1.67 | 1.47 | 1.86 |
| 6 | Other | Q17 | 3.00 | 2.79 | 3.20 |
| 0 | General Physician | Q18 | 2.43 | 2.26 | 2.60 |
| 1 | Specialist Physican | Q18 | 2.53 | 2.38 | 2.69 |
| 2 | Pharmacist | Q18 | 2.59 | 2.43 | 2.76 |
| 3 | Clinical Pharmacist | Q18 | 2.67 | 2.49 | 2.85 |
| 4 | Nurse | Q18 | 2.78 | 2.61 | 2.96 |
| 5 | Midwife | Q18 | 2.00 | 1.84 | 2.16 |
| 6 | Other | Q18 | 2.33 | 2.11 | 2.56 |
| 0 | General Physician | Q19 | 2.64 | 2.48 | 2.82 |
| 1 | Specialist Physican | Q19 | 2.93 | 2.82 | 3.04 |
| 2 | Pharmacist | Q19 | 2.87 | 2.72 | 3.02 |
| 3 | Clinical Pharmacist | Q19 | 2.92 | 2.77 | 3.06 |
| 4 | Nurse | Q19 | 2.69 | 2.51 | 2.85 |
| 5 | Midwife | Q19 | 2.33 | 2.24 | 2.42 |
| 6 | Other | Q19 | 2.93 | 2.75 | 3.14 |
| 0 | General Physician | Q20 | 2.50 | 2.30 | 2.68 |
| 1 | Specialist Physican | Q20 | 2.93 | 2.81 | 3.07 |
| 2 | Pharmacist | Q20 | 2.97 | 2.80 | 3.14 |
| 3 | Clinical Pharmacist | Q20 | 3.04 | 2.88 | 3.18 |
| 4 | Nurse | Q20 | 2.71 | 2.55 | 2.87 |
| 5 | Midwife | Q20 | 2.33 | 2.25 | 2.43 |
| 6 | Other | Q20 | 2.93 | 2.68 | 3.17 |
| 0 | General Physician | Q21 | 2.29 | 2.08 | 2.47 |
| 1 | Specialist Physican | Q21 | 2.80 | 2.60 | 3.00 |
| 2 | Pharmacist | Q21 | 2.88 | 2.72 | 3.03 |
| 3 | Clinical Pharmacist | Q21 | 2.92 | 2.79 | 3.08 |
| 4 | Nurse | Q21 | 2.78 | 2.58 | 2.95 |
| 5 | Midwife | Q21 | 2.33 | 2.25 | 2.42 |
| 6 | Other | Q21 | 3.00 | 2.78 | 3.19 |
| 0 | General Physician | Q22 | 2.43 | 2.26 | 2.60 |
| 1 | Specialist Physican | Q22 | 2.73 | 2.59 | 2.88 |
| 2 | Pharmacist | Q22 | 3.01 | 2.86 | 3.18 |
| 3 | Clinical Pharmacist | Q22 | 3.00 | 2.85 | 3.12 |
| 4 | Nurse | Q22 | 2.78 | 2.59 | 2.95 |
| 5 | Midwife | Q22 | 2.33 | 2.25 | 2.42 |
| 6 | Other | Q22 | 2.87 | 2.64 | 3.07 |
| 0 | General Physician | Q23 | 2.36 | 2.15 | 2.58 |
| 1 | Specialist Physican | Q23 | 2.80 | 2.63 | 2.97 |
| 2 | Pharmacist | Q23 | 2.95 | 2.77 | 3.09 |
| 3 | Clinical Pharmacist | Q23 | 2.94 | 2.79 | 3.09 |
| 4 | Nurse | Q23 | 2.71 | 2.54 | 2.87 |
| 5 | Midwife | Q23 | 2.33 | 2.24 | 2.42 |
| 6 | Other | Q23 | 2.73 | 2.54 | 2.93 |
| 0 | General Physician | Q24 | 2.64 | 2.49 | 2.80 |
| 1 | Specialist Physican | Q24 | 2.53 | 2.38 | 2.68 |
| 2 | Pharmacist | Q24 | 3.01 | 2.86 | 3.17 |
| 3 | Clinical Pharmacist | Q24 | 2.94 | 2.80 | 3.09 |
| 4 | Nurse | Q24 | 2.67 | 2.48 | 2.83 |
| 5 | Midwife | Q24 | 2.00 | 1.85 | 2.16 |
| 6 | Other | Q24 | 2.73 | 2.53 | 2.92 |
| 0 | General Physician | Q25 | 2.43 | 2.25 | 2.61 |
| 1 | Specialist Physican | Q25 | 2.73 | 2.56 | 2.92 |
| 2 | Pharmacist | Q25 | 2.94 | 2.78 | 3.08 |
| 3 | Clinical Pharmacist | Q25 | 2.96 | 2.82 | 3.11 |
| 4 | Nurse | Q25 | 2.79 | 2.64 | 2.97 |
| 5 | Midwife | Q25 | 2.00 | 1.84 | 2.16 |
| 6 | Other | Q25 | 2.80 | 2.58 | 2.99 |
| 0 | General Physician | Q26 | 3.00 | 2.83 | 3.15 |
| 1 | Specialist Physican | Q26 | 2.67 | 2.52 | 2.82 |
| 2 | Pharmacist | Q26 | 2.97 | 2.84 | 3.12 |
| 3 | Clinical Pharmacist | Q26 | 2.92 | 2.77 | 3.06 |
| 4 | Nurse | Q26 | 2.74 | 2.57 | 2.93 |
| 5 | Midwife | Q26 | 2.00 | 1.83 | 2.15 |
| 6 | Other | Q26 | 2.93 | 2.70 | 3.15 |
| 0 | General Physician | Q27 | 2.86 | 2.64 | 3.05 |
| 1 | Specialist Physican | Q27 | 2.80 | 2.65 | 2.95 |
| 2 | Pharmacist | Q27 | 3.08 | 2.89 | 3.24 |
| 3 | Clinical Pharmacist | Q27 | 3.12 | 2.95 | 3.26 |
| 4 | Nurse | Q27 | 2.81 | 2.63 | 2.99 |
| 5 | Midwife | Q27 | 2.00 | 1.83 | 2.16 |
| 6 | Other | Q27 | 2.87 | 2.64 | 3.10 |

| **Table S5.** Comparison between mean knowledge score across job functions using two-sided Wilcoxon Signed-Rank test.^*^ | | | | | | | | |
| --- | --- | --- | --- | --- | --- | --- | --- | --- |
| Q | Job Row | Clinical.Pharmacist | General.Physician | Midwife | Nurse | Other | Pharmacist | Specialist |
| Q15 | Clinical Pharmacist | 1.000 | 0.101 | 0.265 | 0.002 | 0.870 | 0.116 | 0.448 |
| Q15 | General Physician | 0.101 | 1.000 | 0.696 | 0.940 | 0.387 | 0.368 | 0.521 |
| Q15 | Midwife | 0.265 | 0.696 | 1.000 | 0.570 | 0.535 | 0.398 | 0.535 |
| Q15 | Nurse | 0.002 | 0.940 | 0.570 | 1.000 | 0.164 | 0.047 | 0.269 |
| Q15 | Other | 0.870 | 0.387 | 0.535 | 0.164 | 1.000 | 0.511 | 0.709 |
| Q15 | Pharmacist | 0.116 | 0.368 | 0.398 | 0.047 | 0.511 | 1.000 | 0.911 |
| Q15 | Specialist | 0.448 | 0.521 | 0.535 | 0.269 | 0.709 | 0.911 | 1.000 |
| Q16 | Clinical Pharmacist | 1.000 | 0.623 | 0.082 | 0.064 | 0.799 | 0.518 | 0.548 |
| Q16 | General Physician | 0.623 | 1.000 | 0.119 | 0.409 | 0.574 | 0.357 | 0.888 |
| Q16 | Midwife | 0.082 | 0.119 | 1.000 | 0.237 | 0.214 | 0.057 | 0.290 |
| Q16 | Nurse | 0.064 | 0.409 | 0.237 | 1.000 | 0.238 | 0.006 | 0.662 |
| Q16 | Other | 0.799 | 0.574 | 0.214 | 0.238 | 1.000 | 0.928 | 0.615 |
| Q16 | Pharmacist | 0.518 | 0.357 | 0.057 | 0.006 | 0.928 | 1.000 | 0.316 |
| Q16 | Specialist | 0.548 | 0.888 | 0.290 | 0.662 | 0.615 | 0.316 | 1.000 |
| Q17 | Clinical Pharmacist | 1.000 | 0.320 | 0.042 | 0.091 | 0.722 | 0.281 | 0.483 |
| Q17 | General Physician | 0.320 | 1.000 | 0.216 | 0.856 | 0.400 | 0.580 | 0.665 |
| Q17 | Midwife | 0.042 | 0.216 | 1.000 | 0.087 | 0.095 | 0.063 | 0.095 |
| Q17 | Nurse | 0.091 | 0.856 | 0.087 | 1.000 | 0.223 | 0.444 | 0.727 |
| Q17 | Other | 0.722 | 0.400 | 0.095 | 0.223 | 1.000 | 0.385 | 0.557 |
| Q17 | Pharmacist | 0.281 | 0.580 | 0.063 | 0.444 | 0.385 | 1.000 | 0.947 |
| Q17 | Specialist | 0.483 | 0.665 | 0.095 | 0.727 | 0.557 | 0.947 | 1.000 |
| Q18 | Clinical Pharmacist | 1.000 | 0.395 | 0.218 | 0.561 | 0.296 | 0.575 | 0.553 |
| Q18 | General Physician | 0.395 | 1.000 | 0.500 | 0.236 | 0.820 | 0.570 | 0.812 |
| Q18 | Midwife | 0.218 | 0.500 | 1.000 | 0.181 | 0.712 | 0.267 | 0.369 |
| Q18 | Nurse | 0.561 | 0.236 | 0.181 | 1.000 | 0.170 | 0.225 | 0.345 |
| Q18 | Other | 0.296 | 0.820 | 0.712 | 0.170 | 1.000 | 0.381 | 0.617 |
| Q18 | Pharmacist | 0.575 | 0.570 | 0.267 | 0.225 | 0.381 | 1.000 | 0.799 |
| Q18 | Specialist | 0.553 | 0.812 | 0.369 | 0.345 | 0.617 | 0.799 | 1.000 |
| Q19 | Clinical Pharmacist | 1.000 | 0.298 | 0.128 | 0.122 | 0.727 | 0.609 | 0.875 |
| Q19 | General Physician | 0.298 | 1.000 | 0.500 | 0.952 | 0.386 | 0.413 | 0.431 |
| Q19 | Midwife | 0.128 | 0.500 | 1.000 | 0.414 | 0.324 | 0.167 | 0.133 |
| Q19 | Nurse | 0.122 | 0.952 | 0.414 | 1.000 | 0.304 | 0.187 | 0.321 |
| Q19 | Other | 0.727 | 0.386 | 0.324 | 0.304 | 1.000 | 0.572 | 0.739 |
| Q19 | Pharmacist | 0.609 | 0.413 | 0.167 | 0.187 | 0.572 | 1.000 | 0.847 |
| Q19 | Specialist | 0.875 | 0.431 | 0.133 | 0.321 | 0.739 | 0.847 | 1.000 |
| Q20 | Clinical Pharmacist | 1.000 | 0.041 | 0.101 | 0.025 | 0.896 | 0.561 | 0.488 |
| Q20 | General Physician | 0.041 | 1.000 | 0.789 | 0.429 | 0.240 | 0.061 | 0.200 |
| Q20 | Midwife | 0.101 | 0.789 | 1.000 | 0.370 | 0.354 | 0.129 | 0.195 |
| Q20 | Nurse | 0.025 | 0.429 | 0.370 | 1.000 | 0.264 | 0.045 | 0.369 |
| Q20 | Other | 0.896 | 0.240 | 0.354 | 0.264 | 1.000 | 0.703 | 0.679 |
| Q20 | Pharmacist | 0.561 | 0.061 | 0.129 | 0.045 | 0.703 | 1.000 | 0.728 |
| Q20 | Specialist | 0.488 | 0.200 | 0.195 | 0.369 | 0.679 | 0.728 | 1.000 |
| Q21 | Clinical Pharmacist | 1.000 | 0.033 | 0.173 | 0.415 | 0.543 | 0.893 | 0.643 |
| Q21 | General Physician | 0.033 | 1.000 | 1.000 | 0.097 | 0.074 | 0.030 | 0.221 |
| Q21 | Midwife | 0.173 | 1.000 | 1.000 | 0.291 | 0.291 | 0.172 | 0.488 |
| Q21 | Nurse | 0.415 | 0.097 | 0.291 | 1.000 | 0.299 | 0.428 | 0.994 |
| Q21 | Other | 0.543 | 0.074 | 0.291 | 0.299 | 1.000 | 0.434 | 0.570 |
| Q21 | Pharmacist | 0.893 | 0.030 | 0.172 | 0.428 | 0.434 | 1.000 | 0.703 |
| Q21 | Specialist | 0.643 | 0.221 | 0.488 | 0.994 | 0.570 | 0.703 | 1.000 |
| Q22 | Clinical Pharmacist | 1.000 | 0.042 | 0.098 | 0.181 | 1.000 | 0.936 | 0.168 |
| Q22 | General Physician | 0.042 | 1.000 | 0.895 | 0.243 | 0.259 | 0.035 | 0.462 |
| Q22 | Midwife | 0.098 | 0.895 | 1.000 | 0.332 | 0.390 | 0.115 | 0.476 |
| Q22 | Nurse | 0.181 | 0.243 | 0.332 | 1.000 | 0.577 | 0.119 | 0.729 |
| Q22 | Other | 1.000 | 0.259 | 0.390 | 0.577 | 1.000 | 0.928 | 0.574 |
| Q22 | Pharmacist | 0.936 | 0.035 | 0.115 | 0.119 | 0.928 | 1.000 | 0.161 |
| Q22 | Specialist | 0.168 | 0.462 | 0.476 | 0.729 | 0.574 | 0.161 | 1.000 |
| Q23 | Clinical Pharmacist | 1.000 | 0.087 | 0.117 | 0.158 | 0.661 | 0.991 | 0.552 |
| Q23 | General Physician | 0.087 | 1.000 | 0.947 | 0.333 | 0.382 | 0.074 | 0.332 |
| Q23 | Midwife | 0.117 | 0.947 | 1.000 | 0.373 | 0.458 | 0.158 | 0.342 |
| Q23 | Nurse | 0.158 | 0.333 | 0.373 | 1.000 | 0.777 | 0.122 | 0.748 |
| Q23 | Other | 0.661 | 0.382 | 0.458 | 0.777 | 1.000 | 0.605 | 1.000 |
| Q23 | Pharmacist | 0.991 | 0.074 | 0.158 | 0.122 | 0.605 | 1.000 | 0.555 |
| Q23 | Specialist | 0.552 | 0.332 | 0.342 | 0.748 | 1.000 | 0.555 | 1.000 |
| Q24 | Clinical Pharmacist | 1.000 | 0.185 | 0.072 | 0.120 | 0.661 | 0.495 | 0.057 |
| Q24 | General Physician | 0.185 | 1.000 | 0.316 | 0.857 | 0.680 | 0.100 | 0.691 |
| Q24 | Midwife | 0.072 | 0.316 | 1.000 | 0.246 | 0.293 | 0.066 | 0.410 |
| Q24 | Nurse | 0.120 | 0.857 | 0.246 | 1.000 | 0.720 | 0.023 | 0.508 |
| Q24 | Other | 0.661 | 0.680 | 0.293 | 0.720 | 1.000 | 0.423 | 0.474 |
| Q24 | Pharmacist | 0.495 | 0.100 | 0.066 | 0.023 | 0.423 | 1.000 | 0.028 |
| Q24 | Specialist | 0.057 | 0.691 | 0.410 | 0.508 | 0.474 | 0.028 | 1.000 |
| Q25 | Clinical Pharmacist | 1.000 | 0.034 | 0.072 | 0.281 | 0.840 | 0.859 | 0.237 |
| Q25 | General Physician | 0.034 | 1.000 | 0.550 | 0.148 | 0.265 | 0.036 | 0.439 |
| Q25 | Midwife | 0.072 | 0.550 | 1.000 | 0.152 | 0.233 | 0.081 | 0.276 |
| Q25 | Nurse | 0.281 | 0.148 | 0.152 | 1.000 | 0.736 | 0.301 | 0.627 |
| Q25 | Other | 0.840 | 0.265 | 0.233 | 0.736 | 1.000 | 0.885 | 0.681 |
| Q25 | Pharmacist | 0.859 | 0.036 | 0.081 | 0.301 | 0.885 | 1.000 | 0.265 |
| Q25 | Specialist | 0.237 | 0.439 | 0.276 | 0.627 | 0.681 | 0.265 | 1.000 |
| Q26 | Clinical Pharmacist | 1.000 | 0.580 | 0.075 | 0.295 | 0.513 | 0.691 | 0.167 |
| Q26 | General Physician | 0.580 | 1.000 | 0.122 | 0.298 | 0.908 | 0.741 | 0.221 |
| Q26 | Midwife | 0.075 | 0.122 | 1.000 | 0.199 | 0.194 | 0.068 | 0.304 |
| Q26 | Nurse | 0.295 | 0.298 | 0.199 | 1.000 | 0.333 | 0.127 | 0.619 |
| Q26 | Other | 0.513 | 0.908 | 0.194 | 0.333 | 1.000 | 0.650 | 0.341 |
| Q26 | Pharmacist | 0.691 | 0.741 | 0.068 | 0.127 | 0.650 | 1.000 | 0.110 |
| Q26 | Specialist | 0.167 | 0.221 | 0.304 | 0.619 | 0.341 | 0.110 | 1.000 |
| Q27 | Clinical Pharmacist | 1.000 | 0.499 | 0.050 | 0.065 | 0.658 | 0.795 | 0.127 |
| Q27 | General Physician | 0.499 | 1.000 | 0.241 | 0.777 | 0.945 | 0.563 | 0.749 |
| Q27 | Midwife | 0.050 | 0.241 | 1.000 | 0.172 | 0.243 | 0.052 | 0.207 |
| Q27 | Nurse | 0.065 | 0.777 | 0.172 | 1.000 | 0.674 | 0.060 | 0.886 |
| Q27 | Other | 0.658 | 0.945 | 0.243 | 0.674 | 1.000 | 0.741 | 0.665 |
| Q27 | Pharmacist | 0.795 | 0.563 | 0.052 | 0.060 | 0.741 | 1.000 | 0.142 |
| Q27 | Specialist | 0.127 | 0.749 | 0.207 | 0.886 | 0.665 | 0.142 | 1.000 |
| ^*^Highlighted cells represent significant P-value. | | | | | | | | |

| **Table S6.** Mean practice scores across job functions. | | | | | |
| --- | --- | --- | --- | --- | --- |
| JOB | Job Function | Q | mean | LCL | UCL |
| 0 | General Physician | Q28 | 3.86 | 3.73 | 3.99 |
| 1 | Specialist Physican | Q28 | 3.80 | 3.68 | 3.93 |
| 2 | Pharmacist | Q28 | 3.80 | 3.68 | 3.93 |
| 3 | Clinical Pharmacist | Q28 | 3.88 | 3.76 | 4.01 |
| 4 | Nurse | Q28 | 3.60 | 3.40 | 3.78 |
| 5 | Midwife | Q28 | 4.33 | 4.24 | 4.43 |
| 6 | Other | Q28 | 3.33 | 3.13 | 3.54 |
| 0 | General Physician | Q29 | 4.07 | 3.94 | 4.21 |
| 1 | Specialist Physican | Q29 | 3.67 | 3.56 | 3.76 |
| 2 | Pharmacist | Q29 | 3.88 | 3.75 | 4.02 |
| 3 | Clinical Pharmacist | Q29 | 3.88 | 3.76 | 3.99 |
| 4 | Nurse | Q29 | 3.64 | 3.45 | 3.82 |
| 5 | Midwife | Q29 | 4.67 | 4.57 | 4.76 |
| 6 | Other | Q29 | 3.60 | 3.41 | 3.81 |
| 0 | General Physician | Q30 | 3.86 | 3.69 | 4.01 |
| 1 | Specialist Physican | Q30 | 3.80 | 3.70 | 3.90 |
| 2 | Pharmacist | Q30 | 3.74 | 3.60 | 3.89 |
| 3 | Clinical Pharmacist | Q30 | 3.94 | 3.81 | 4.07 |
| 4 | Nurse | Q30 | 3.69 | 3.48 | 3.86 |
| 5 | Midwife | Q30 | 4.33 | 4.24 | 4.43 |
| 6 | Other | Q30 | 3.40 | 3.18 | 3.62 |
| 0 | General Physician | Q31 | 3.93 | 3.74 | 4.08 |
| 1 | Specialist Physican | Q31 | 3.80 | 3.68 | 3.92 |
| 2 | Pharmacist | Q31 | 3.84 | 3.68 | 4.00 |
| 3 | Clinical Pharmacist | Q31 | 3.94 | 3.81 | 4.06 |
| 4 | Nurse | Q31 | 3.66 | 3.45 | 3.85 |
| 5 | Midwife | Q31 | 4.33 | 4.25 | 4.44 |
| 6 | Other | Q31 | 3.67 | 3.46 | 3.88 |
| 0 | General Physician | Q32 | 3.64 | 3.45 | 3.82 |
| 1 | Specialist Physican | Q32 | 4.00 | 3.89 | 4.10 |
| 2 | Pharmacist | Q32 | 3.83 | 3.68 | 3.97 |
| 3 | Clinical Pharmacist | Q32 | 3.82 | 3.67 | 3.97 |
| 4 | Nurse | Q32 | 3.55 | 3.35 | 3.76 |
| 5 | Midwife | Q32 | 4.67 | 4.56 | 4.77 |
| 6 | Other | Q32 | 3.73 | 3.53 | 3.95 |
| 0 | General Physician | Q33 | 3.71 | 3.51 | 3.94 |
| 1 | Specialist Physican | Q33 | 3.60 | 3.44 | 3.77 |
| 2 | Pharmacist | Q33 | 3.80 | 3.64 | 3.96 |
| 3 | Clinical Pharmacist | Q33 | 3.76 | 3.58 | 3.95 |
| 4 | Nurse | Q33 | 3.72 | 3.49 | 3.91 |
| 5 | Midwife | Q33 | 5.00 | 5.00 | 5.00 |
| 6 | Other | Q33 | 3.60 | 3.40 | 3.80 |
| 0 | General Physician | Q34 | 3.71 | 3.56 | 3.87 |
| 1 | Specialist Physican | Q34 | 3.80 | 3.68 | 3.92 |
| 2 | Pharmacist | Q34 | 3.67 | 3.52 | 3.82 |
| 3 | Clinical Pharmacist | Q34 | 3.92 | 3.79 | 4.06 |
| 4 | Nurse | Q34 | 3.53 | 3.33 | 3.74 |
| 5 | Midwife | Q34 | 4.67 | 4.58 | 4.76 |
| 6 | Other | Q34 | 3.60 | 3.41 | 3.77 |
| 0 | General Physician | Q35 | 3.71 | 3.56 | 3.87 |
| 1 | Specialist Physican | Q35 | 3.80 | 3.69 | 3.92 |
| 2 | Pharmacist | Q35 | 3.77 | 3.61 | 3.92 |
| 3 | Clinical Pharmacist | Q35 | 3.84 | 3.68 | 3.99 |
| 4 | Nurse | Q35 | 3.60 | 3.40 | 3.80 |
| 5 | Midwife | Q35 | 4.67 | 4.58 | 4.76 |
| 6 | Other | Q35 | 3.73 | 3.55 | 3.90 |
| 0 | General Physician | Q36 | 3.93 | 3.80 | 4.07 |
| 1 | Specialist Physican | Q36 | 3.93 | 3.86 | 4.01 |
| 2 | Pharmacist | Q36 | 3.90 | 3.75 | 4.05 |
| 3 | Clinical Pharmacist | Q36 | 4.00 | 3.87 | 4.12 |
| 4 | Nurse | Q36 | 3.60 | 3.41 | 3.79 |
| 5 | Midwife | Q36 | 5.00 | 5.00 | 5.00 |
| 6 | Other | Q36 | 3.80 | 3.56 | 4.04 |
| 0 | General Physician | Q37 | 3.71 | 3.54 | 3.89 |
| 1 | Specialist Physican | Q37 | 3.93 | 3.82 | 4.04 |
| 2 | Pharmacist | Q37 | 3.74 | 3.58 | 3.88 |
| 3 | Clinical Pharmacist | Q37 | 4.02 | 3.89 | 4.15 |
| 4 | Nurse | Q37 | 3.53 | 3.32 | 3.72 |
| 5 | Midwife | Q37 | 5.00 | 5.00 | 5.00 |
| 6 | Other | Q37 | 3.87 | 3.68 | 4.05 |
| 0 | General Physician | Q38 | 3.93 | 3.77 | 4.07 |
| 1 | Specialist Physican | Q38 | 3.80 | 3.67 | 3.92 |
| 2 | Pharmacist | Q38 | 3.76 | 3.62 | 3.90 |
| 3 | Clinical Pharmacist | Q38 | 3.92 | 3.76 | 4.06 |
| 4 | Nurse | Q38 | 3.50 | 3.31 | 3.70 |
| 5 | Midwife | Q38 | 4.67 | 4.56 | 4.75 |
| 6 | Other | Q38 | 3.80 | 3.63 | 3.98 |
| 0 | General Physician | Q39 | 3.93 | 3.79 | 4.06 |
| 1 | Specialist Physican | Q39 | 3.53 | 3.37 | 3.71 |
| 2 | Pharmacist | Q39 | 3.63 | 3.47 | 3.79 |
| 3 | Clinical Pharmacist | Q39 | 4.00 | 3.85 | 4.14 |
| 4 | Nurse | Q39 | 3.62 | 3.42 | 3.83 |
| 5 | Midwife | Q39 | 5.00 | 5.00 | 5.00 |
| 6 | Other | Q39 | 3.67 | 3.43 | 3.87 |

| **Table S7.** Comparison between mean practice score across job functions using two-sided Wilcoxon Signed-Rank test.^*^ | | | | | | | | |
| --- | --- | --- | --- | --- | --- | --- | --- | --- |
| Q | Job Row | Clinical.Pharmacist | General.Physician | Midwife | Nurse | Other | Pharmacist | Specialist |
| Q28 | Clinical Pharmacist | 1.000 | 0.774 | 0.256 | 0.214 | 0.027 | 0.449 | 0.542 |
| Q28 | General Physician | 0.774 | 1.000 | 0.285 | 0.608 | 0.120 | 0.887 | 0.826 |
| Q28 | Midwife | 0.256 | 0.285 | 1.000 | 0.172 | 0.117 | 0.181 | 0.235 |
| Q28 | Nurse | 0.214 | 0.608 | 0.172 | 1.000 | 0.174 | 0.482 | 0.809 |
| Q28 | Other | 0.027 | 0.120 | 0.117 | 0.174 | 1.000 | 0.041 | 0.157 |
| Q28 | Pharmacist | 0.449 | 0.887 | 0.181 | 0.482 | 0.041 | 1.000 | 0.860 |
| Q28 | Specialist | 0.542 | 0.826 | 0.235 | 0.809 | 0.157 | 0.860 | 1.000 |
| Q29 | Clinical Pharmacist | 1.000 | 0.351 | 0.027 | 0.233 | 0.229 | 0.947 | 0.158 |
| Q29 | General Physician | 0.351 | 1.000 | 0.219 | 0.141 | 0.189 | 0.388 | 0.113 |
| Q29 | Midwife | 0.027 | 0.219 | 1.000 | 0.039 | 0.096 | 0.047 | 0.020 |
| Q29 | Nurse | 0.233 | 0.141 | 0.039 | 1.000 | 0.717 | 0.171 | 0.736 |
| Q29 | Other | 0.229 | 0.189 | 0.096 | 0.717 | 1.000 | 0.218 | 0.838 |
| Q29 | Pharmacist | 0.947 | 0.388 | 0.047 | 0.171 | 0.218 | 1.000 | 0.175 |
| Q29 | Specialist | 0.158 | 0.113 | 0.020 | 0.736 | 0.838 | 0.175 | 1.000 |
| Q30 | Clinical Pharmacist | 1.000 | 0.598 | 0.306 | 0.214 | 0.070 | 0.136 | 0.385 |
| Q30 | General Physician | 0.598 | 1.000 | 0.342 | 0.781 | 0.303 | 0.791 | 0.922 |
| Q30 | Midwife | 0.306 | 0.342 | 1.000 | 0.216 | 0.198 | 0.158 | 0.177 |
| Q30 | Nurse | 0.214 | 0.781 | 0.216 | 1.000 | 0.318 | 0.978 | 0.928 |
| Q30 | Other | 0.070 | 0.303 | 0.198 | 0.318 | 1.000 | 0.223 | 0.317 |
| Q30 | Pharmacist | 0.136 | 0.791 | 0.158 | 0.978 | 0.223 | 1.000 | 0.927 |
| Q30 | Specialist | 0.385 | 0.922 | 0.177 | 0.928 | 0.317 | 0.927 | 1.000 |
| Q31 | Clinical Pharmacist | 1.000 | 0.887 | 0.330 | 0.177 | 0.364 | 0.520 | 0.415 |
| Q31 | General Physician | 0.887 | 1.000 | 0.461 | 0.516 | 0.596 | 0.854 | 0.707 |
| Q31 | Midwife | 0.330 | 0.461 | 1.000 | 0.205 | 0.320 | 0.252 | 0.235 |
| Q31 | Nurse | 0.177 | 0.516 | 0.205 | 1.000 | 0.936 | 0.360 | 0.882 |
| Q31 | Other | 0.364 | 0.596 | 0.320 | 0.936 | 1.000 | 0.533 | 0.825 |
| Q31 | Pharmacist | 0.520 | 0.854 | 0.252 | 0.360 | 0.533 | 1.000 | 0.662 |
| Q31 | Specialist | 0.415 | 0.707 | 0.235 | 0.882 | 0.825 | 0.662 | 1.000 |
| Q32 | Clinical Pharmacist | 1.000 | 0.351 | 0.057 | 0.165 | 0.821 | 0.918 | 0.537 |
| Q32 | General Physician | 0.351 | 1.000 | 0.098 | 0.982 | 0.663 | 0.317 | 0.206 |
| Q32 | Midwife | 0.057 | 0.098 | 1.000 | 0.060 | 0.153 | 0.047 | 0.075 |
| Q32 | Nurse | 0.165 | 0.982 | 0.060 | 1.000 | 0.553 | 0.115 | 0.132 |
| Q32 | Other | 0.821 | 0.663 | 0.153 | 0.553 | 1.000 | 0.829 | 0.538 |
| Q32 | Pharmacist | 0.918 | 0.317 | 0.047 | 0.115 | 0.829 | 1.000 | 0.453 |
| Q32 | Specialist | 0.537 | 0.206 | 0.075 | 0.132 | 0.538 | 0.453 | 1.000 |
| Q33 | Clinical Pharmacist | 1.000 | 1.000 | 0.008 | 0.911 | 0.464 | 0.937 | 0.477 |
| Q33 | General Physician | 1.000 | 1.000 | 0.081 | 0.988 | 0.838 | 0.944 | 0.731 |
| Q33 | Midwife | 0.008 | 0.081 | NA | 0.019 | 0.043 | 0.007 | 0.009 |
| Q33 | Nurse | 0.911 | 0.988 | 0.019 | 1.000 | 0.581 | 0.921 | 0.590 |
| Q33 | Other | 0.464 | 0.838 | 0.043 | 0.581 | 1.000 | 0.425 | 0.860 |
| Q33 | Pharmacist | 0.937 | 0.944 | 0.007 | 0.921 | 0.425 | 1.000 | 0.494 |
| Q33 | Specialist | 0.477 | 0.731 | 0.009 | 0.590 | 0.860 | 0.494 | 1.000 |
| Q34 | Clinical Pharmacist | 1.000 | 0.266 | 0.068 | 0.042 | 0.139 | 0.077 | 0.486 |
| Q34 | General Physician | 0.266 | 1.000 | 0.094 | 0.855 | 0.760 | 0.839 | 0.671 |
| Q34 | Midwife | 0.068 | 0.094 | 1.000 | 0.035 | 0.081 | 0.026 | 0.071 |
| Q34 | Nurse | 0.042 | 0.855 | 0.035 | 1.000 | 0.873 | 0.510 | 0.474 |
| Q34 | Other | 0.139 | 0.760 | 0.081 | 0.873 | 1.000 | 0.542 | 0.463 |
| Q34 | Pharmacist | 0.077 | 0.839 | 0.026 | 0.510 | 0.542 | 1.000 | 0.707 |
| Q34 | Specialist | 0.486 | 0.671 | 0.071 | 0.474 | 0.463 | 0.707 | 1.000 |
| Q35 | Clinical Pharmacist | 1.000 | 0.376 | 0.069 | 0.248 | 0.551 | 0.555 | 0.622 |
| Q35 | General Physician | 0.376 | 1.000 | 0.094 | 0.958 | 0.944 | 0.492 | 0.671 |
| Q35 | Midwife | 0.069 | 0.094 | 1.000 | 0.052 | 0.136 | 0.038 | 0.071 |
| Q35 | Nurse | 0.248 | 0.958 | 0.052 | 1.000 | 0.874 | 0.414 | 0.724 |
| Q35 | Other | 0.551 | 0.944 | 0.136 | 0.874 | 1.000 | 0.705 | 0.825 |
| Q35 | Pharmacist | 0.555 | 0.492 | 0.038 | 0.414 | 0.705 | 1.000 | 0.861 |
| Q35 | Specialist | 0.622 | 0.671 | 0.071 | 0.724 | 0.825 | 0.861 | 1.000 |
| Q36 | Clinical Pharmacist | 1.000 | 0.656 | 0.006 | 0.051 | 0.856 | 0.564 | 0.617 |
| Q36 | General Physician | 0.656 | 1.000 | 0.031 | 0.416 | 0.982 | 0.931 | 0.959 |
| Q36 | Midwife | 0.006 | 0.031 | NA | 0.012 | 0.068 | 0.011 | 0.004 |
| Q36 | Nurse | 0.051 | 0.416 | 0.012 | 1.000 | 0.432 | 0.109 | 0.329 |
| Q36 | Other | 0.856 | 0.982 | 0.068 | 0.432 | 1.000 | 0.965 | 0.908 |
| Q36 | Pharmacist | 0.564 | 0.931 | 0.011 | 0.109 | 0.965 | 1.000 | 0.956 |
| Q36 | Specialist | 0.617 | 0.959 | 0.004 | 0.329 | 0.908 | 0.956 | 1.000 |
| Q37 | Clinical Pharmacist | 1.000 | 0.215 | 0.016 | 0.014 | 0.585 | 0.044 | 0.597 |
| Q37 | General Physician | 0.215 | 1.000 | 0.034 | 0.708 | 0.646 | 0.772 | 0.474 |
| Q37 | Midwife | 0.016 | 0.034 | NA | 0.011 | 0.045 | 0.003 | 0.012 |
| Q37 | Nurse | 0.014 | 0.708 | 0.011 | 1.000 | 0.317 | 0.265 | 0.208 |
| Q37 | Other | 0.585 | 0.646 | 0.045 | 0.317 | 1.000 | 0.608 | 0.927 |
| Q37 | Pharmacist | 0.044 | 0.772 | 0.003 | 0.265 | 0.608 | 1.000 | 0.438 |
| Q37 | Specialist | 0.597 | 0.474 | 0.012 | 0.208 | 0.927 | 0.438 | 1.000 |
| Q38 | Clinical Pharmacist | 1.000 | 0.861 | 0.083 | 0.020 | 0.568 | 0.152 | 0.395 |
| Q38 | General Physician | 0.861 | 1.000 | 0.182 | 0.202 | 0.765 | 0.582 | 0.707 |
| Q38 | Midwife | 0.083 | 0.182 | 1.000 | 0.049 | 0.153 | 0.041 | 0.071 |
| Q38 | Nurse | 0.020 | 0.202 | 0.049 | 1.000 | 0.376 | 0.120 | 0.353 |
| Q38 | Other | 0.568 | 0.765 | 0.153 | 0.376 | 1.000 | 0.923 | 0.982 |
| Q38 | Pharmacist | 0.152 | 0.582 | 0.041 | 0.120 | 0.923 | 1.000 | 0.988 |
| Q38 | Specialist | 0.395 | 0.707 | 0.071 | 0.353 | 0.982 | 0.988 | 1.000 |
| Q39 | Clinical Pharmacist | 1.000 | 0.702 | 0.021 | 0.067 | 0.287 | 0.015 | 0.080 |
| Q39 | General Physician | 0.702 | 1.000 | 0.031 | 0.399 | 0.563 | 0.285 | 0.292 |
| Q39 | Midwife | 0.021 | 0.031 | NA | 0.014 | 0.045 | 0.004 | 0.010 |
| Q39 | Nurse | 0.067 | 0.399 | 0.014 | 1.000 | 0.932 | 0.900 | 0.712 |
| Q39 | Other | 0.287 | 0.563 | 0.045 | 0.932 | 1.000 | 0.908 | 0.774 |
| Q39 | Pharmacist | 0.015 | 0.285 | 0.004 | 0.900 | 0.908 | 1.000 | 0.732 |
| Q39 | Specialist | 0.080 | 0.292 | 0.010 | 0.712 | 0.774 | 0.732 | 1.000 |
| ^*^Highlighted cells represent significant P-value. | | | | | | | | |

**Appendix 1: study survey**

**National Pharmacovigilance Program 2022**

***Evaluation Form***

Dear Health Care Provider,

You are kindly asked to fill this survey questionnaire as a follow up to your participation in one of the pharmacovigilance awareness workshops held by Jordan Food and Drug administration (JFDA) in collaboration with WHO Jordan country office last year; as part of the national pharmacovigilance building capacity program.

The survey aims to evaluate the improvement in your knowledge of Pharmacovigilance post attending the workshop, to evaluate the pharmacovigilance building capacity program (materials and speakers) and to evaluate the impact of this program on Jordan’s regional pharmacovigilance centers; as there were also attendees from these centers (King Abdullah University Hospital, Al-Basheer Hospital, Prince Hamzah Hospital, Jordan University Hospital and The Royal Medical Services).

The questionnaire consists of 4 sections, varying between “Tick the most appropriate answer” questions, “Strongly agree to strongly disagree” evaluation score questions and editorial questions, it will take less than 15 minutes to be filled.

Sections B and C are to be completed by healthcare practitioners (HCPs).

Information provided will be handled confidentially and are solely used for the sake of the survey.

Your cooperation & timely feedback is highly appreciated.

Thank you!

**A – Background Information**

**Q1. Please tick ONE answer that best describes your current primary position.**

- General Physician
- Specialist Physician
- Pharmacist
- Clinical Pharmacist
- Nurse
- Midwife
- Other (please specify: _______________

**Q2. Gender identity?**

- Male
- Female

**Q3. What is your age group?**

- 18 to 24
- 25 to 34
- 35 to 44
- 45 to 54
- 55 or over

**Q4. How many years of work experience do you have?**

- None
- two years or less
- 3 to 5 years
- 6 to 10 years
- 11 to 15 years
- 16 to 20 years
- 21 to 25 years
- 26 or over

**Q5. What is the highest level of education that you have completed?**

- One Bachelor’s Degree
- Two Bachelor’s Degrees
- One Master’s Degree
- Two Master’s Degrees
- PhD
- Professional Certification from an accredited educational institution
- Other (please specify: _______________

**Q6. Are you a regional center (focal point, staff) following the national center at JFDA?**

- Yes
- No

**B – Trainers Evaluation:**

|  |  |
| --- | --- |

1 2 3 4 5

**Unacceptable Needs Meets Exceeds Outstanding**

**Improvement Expectations Expectations**

| **No.** | **Questions** | **Score** |
| --- | --- | --- |
| **Q7** | How do evaluate trainers in relation to their technical knowledge |  |
| **Q8** | How do you evaluate trainers in relation to effective verbal communication? |  |
| **Q9** | After enrollment in the national pharmacovigilance program training session, how do you evaluate this program as training of trainer? |  |
|  | **Total score** |  |

**C – Training Evaluation**

**Q10.** What are the most important **things you learned** during this training?

***(You can choose more than one choice)***

- Technical information
- Application of skills in the field
- Team building
- Communication
- Others (please specify) ………………………………………………….

**Q11.** What are the most remarkable strengths of the training program?

***(You can choose more than one choice)***

- Facilitation, training materials
- Participant selection
- Technical area of training
- Place of activity conducted
- Others (please specify) ………………………………………………….

**Q12.** What presentation styles/Methodology of the training were the most effective for you?

***(You can choose more than one choice)***

- Case studies
- Role play
- Lectures
- Quizzes
- Group exercises
- Others (please specify) …………………………………………………..

**Q13.** Please provide up to two examples of how your practices have been changed due to this training program (if any).

**Q14.** What post-training support might you like to implement what you’ve learned in this training program?

- Supervisory support
- Videos
- Newsletters
- Preceptorships
- Clinical consultations
- Facility-based training,
- Others (Specify) …………………………….

Please rate the improvement in your pharmacovigilance knowledge **post-training** in the following areas using the scale below.

|  |  |
| --- | --- |

1 2 3 4

**Stayed the same Slightly Improved Improved Well improved**

| **No.** | **Statements evaluating trainees’ pharmacovigilance knowledge after attending the workshop** | **1** | **2** | **3** | **4** |
| --- | --- | --- | --- | --- | --- |
| **Q15** | How would you rate the level of improvement in your familiarity with the term "Pharmacovigilance (PV)"? |  |  |  |  |
| **Q16** | How would you rate the level of improvement in your belief that “pharmacovigilance is an essential component of the medication life cycle”? |  |  |  |  |
| **Q17** | How would you rate the level of improvement in your familiarity with the WHO’s Program for International Drug Monitoring (PIDM)? |  |  |  |  |
| **Q18** | How would you rate the level of improvement in your familiarity with the Uppsala monitoring center? |  |  |  |  |
| **Q19** | How would you rate the level of improvement in your familiarity with the established national PV center in Jordan? |  |  |  |  |
| **Q20** | How would you rate the level of improvement in your awareness that some ADRs can occur even after years of stopping the product and in next generations (Delayed ADRs)? |  |  |  |  |
| **Q21** | How would you rate the level of improvement in your awareness of the available channels of submitting suspected ADRs reports to the national PV center (e.g., yellow card (paper form), email, FAX, Telephone, Website, QR Code)? |  |  |  |  |
| **Q22** | How would you rate the level of improvement in your awareness that you can report ADRs in both Arabic and English Languages, as reporting forms support these languages? |  |  |  |  |
| **Q23** | How would you rate the level of improvement in your familiarity with the use of ADRs reports and the actions can be made based on the analysis of these collected and validated ones? |  |  |  |  |
| **Q24** | How would you rate the level of improvement in your awareness that by regulation, as a healthcare provider, you are responsible of reporting ADRs? |  |  |  |  |
| **Q25** | How would you rate the level of improvement in your awareness that anyone can directly report ADRs including patients, parents and carers, even you as a health care practitioner, without seeking any higher approvals? |  |  |  |  |
| **Q26** | How would you rate the level of improvement in your belief that reporting suspected ADR to the national PV system may have an influence on the patient's personal, social, and/or economic quality of life? |  |  |  |  |
| **Q27** | How would you rate the level of improvement in your belief that reporting ADRs will help with generating more evidence to ensure better understanding and improvement of the safety profile of a product? |  |  |  |  |

Please rate the improvement in the **practice** of pharmacovigilance **after attending the workshop**

1. Greatly decreased
2. Decreased
3. Stayed the same
4. Increased
5. Greatly increased

| **Please rate the improvement in the *practice* of pharmacovigilance after attending the workshop** | **1** | **2** | **3** | **4** | **5** |
| --- | --- | --- | --- | --- | --- |
| **Q28.** Reporting of suspected ADRs even if you are not sure if the medicine is responsible for the reaction |  |  |  |  |  |
| **Q29.** Reporting of serious and non-serious ADRs |  |  |  |  |  |
| **Q30.** Reporting expected ADRs (known and listed in the product’s leaflet) |  |  |  |  |  |
| **Q31.** Reporting unexpected ADRs (unknown ones, not listed in the product’s leaflet) |  |  |  |  |  |
| **Q32.** Using electronic forms to report ADRs |  |  |  |  |  |
| **Q33.** Using different available channels (e.g., yellow card (paper form), email, FAX, Telephone, Website, QR Code) to report ADRs to the national PV center |  |  |  |  |  |
| **Q34.** Frequency of filing up a suspected ADR form |  |  |  |  |  |
| **Q35.** Easiness of filling up ADR form |  |  |  |  |  |
| **Q36.** Application of your knowledge to recognize suspected ADR during practice and regular interactions with patients |  |  |  |  |  |
| **Q37.** Reading ADR-related articles |  |  |  |  |  |
| **Q38.** Attending training program on ADR reporting |  |  |  |  |  |
| **Q39.** Carrying out research activities related to pharmacovigilance |  |  |  |  |  |

Please rate the training in terms of its impact and usefulness in the following areas using the scale below.

|  |  |
| --- | --- |

1 2 3 4 5

**Strongly Disagree Neutral Agree Strongly**

**Disagree Agree**

| **No.** | **Area/Observation** | **1** | **2** | **3** | **4** | **5** |
| --- | --- | --- | --- | --- | --- | --- |
| **Q40** | The **knowledge and skills** I learned will be helpful to me in my work |  |  |  |  |  |
| **Q41** | The workshop enhanced my **willingness** to provide training and mentor others |  |  |  |  |  |
| **Q42** | The workshop improved my **capacity** to provide training and mentor others |  |  |  |  |  |
| **Q43** | The training/workshop **learning** **objectives** were stated clearly and successfully met |  |  |  |  |  |
| **Q44** | The training/workshop was **well** **facilitated** |  |  |  |  |  |
| **Q45** | The answers the facilitator gave to participants’ questions were **clear and satisfactory** |  |  |  |  |  |
| **Q46** | The facilitator provided illustrative **examples** |  |  |  |  |  |
| **Q47** | The facilitator’s made the **best use of the time** allotted to each session. |  |  |  |  |  |
| **Q48** | I had ample opportunity to ask questions and receive answers to my questions during the training/workshop. |  |  |  |  |  |
| **Q49** | The training/workshop allowed participants to practice **practical skills** related to essential concepts. |  |  |  |  |  |
| **Q50** | The training/workshop was interactive and allowed me to be **actively engaged.** |  |  |  |  |  |
| **Q51** | The workshop was **well organized.** |  |  |  |  |  |
| **Q52** | I found the **venue and set-up** to be comfortable, free of distractions, and conducive to learning. |  |  |  |  |  |
| **Q53** | I would **recommend** this training/workshop to other |  |  |  |  |  |
| **Q54** | I **enjoyed** the workshop. |  |  |  |  |  |

**Q55.** What three areas of improvement would you like to suggest to enhance the training programme? Any other comments?

**Q56.** What is your overall evaluation of the training programme?


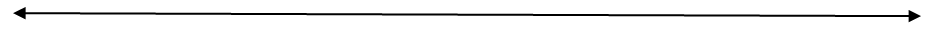


**(Strongly Negative / Negative / Neutral / Positive / Strongly Positive)**
